# Report of a magpie preying on a post‐fledgling Daurian redstart

**DOI:** 10.1002/ece3.10412

**Published:** 2023-08-09

**Authors:** Guofen Zhu, Meinuo Zheng, Shanshan Lyu, Laikun Ma

**Affiliations:** ^1^ Heibei University Baoding China; ^2^ Key Laboratory of Botany State Ethnic Affairs Commission Hebei Normal University for Nationalities Chengde China

**Keywords:** fledgling, *Phoenicurus auroreus*, *Pica pica*, predation

## Abstract

A magpie (*Pica pica*) preying on a fledgling of Daurian redstart (*Phoenicurus auroreus*) was incidentally recorded with a video shot by mobile phone on 26 May 2021, providing direct evidence for magpie predation. It also shows that predation is an important factor that affects the survival of fledglings, indicating that survival of fledglings should be considered in evaluating breeding success of birds. The fledgling was about 13‐day‐old posthatching, and it was on its first day of leaving the nest when the incident occurred. It was preyed upon by a magpie 10 m away from the nest by two attempts under strong defensive behaviour from the female.

## Graphical abstract

This study reports the case of a magpie predating on a fledgling of Daurian redstart with the female parent giving a fierce attack. Our results provided direct evidence that predation is an important threat to the survival of fledglings during the fledging period. And aggressive defensive behaviour exists in the female of Daurian redstarts.

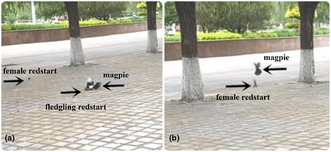

## 1 INTRODUCTION

Predation is the main factor that affects the reproductive performance of birds, and successful predation not only directly results in the loss of eggs or chicks in nests but also threatens the safety of parents and significantly reduces the fitness of birds (Chalfoun et al., 2002; Martin, 1993, 1995; Roos et al., 2018). The types of predator recorded were mainly affected by geographical areas and habitat types, including small mammals (e.g. Fu et al., 2016; Ma et al., 2021), birds (e.g. Deng & Gao, 2005; Fu et al., 2016; Walankiewicz, 2002; Wan et al., 2023; Yin et al., 2023), snakes (e.g. Gartner & Greene, 2008; Ma et al., 2021), insects (e.g. Drees, 1994; Fu et al., 2016) and crabs (Yang et al., 2014).

Bird chicks need to go through two stages between hatching and independent for altricial birds. The nestling period refers to when chicks remain in the breeding nest and receive full parental care. During the post‐fledging period, chicks can leave the breeding nest following their parents, but are unable to survive on their own and still depend on their parents for information on food and predator risks (Jones et al., 2020; Zheng, 2012). The post‐fledging period is a critical transitional stage for independent survival of altricial birds. However, previous studies on nest predation mainly focussed on stage of the nestling in the nest (e.g. Fu et al., 2016; Guppy et al., 2017; Yin et al., 2023), and research on the post‐fledging period is relatively rare and difficult because of moving about. In addition, compared with adult birds, predation remains a major risk to the survival of fledglings at this stage, as they are relatively weak in avoiding risks and self‐protection (Haché et al., 2014; King et al., 2006; Sullivan, 1989).

The Daurian redstart (*Phoenicurus auroreus*) is a small bird of the order Passeriformes, a secondary nesting bird that breeds in the cracks of house walls, cracks between the door and its frame, house piers, rocky crevices in roads, ridges on forest edges, pits of stone ridges and haystacks and on trees. Magpies (*Pica pica*) are large, omnivorous birds of the order Passeriformes, which prey on the eggs or chicks of other birds during the breeding season (Bravo et al., 2020; Kryštofková et al., 2011; Zhao, 2001). This study records a case of a magpie preying on a post‐fledgling of Daurian redstart.

## 2 METHODS AND RESULTS

**FIGURE 1:**
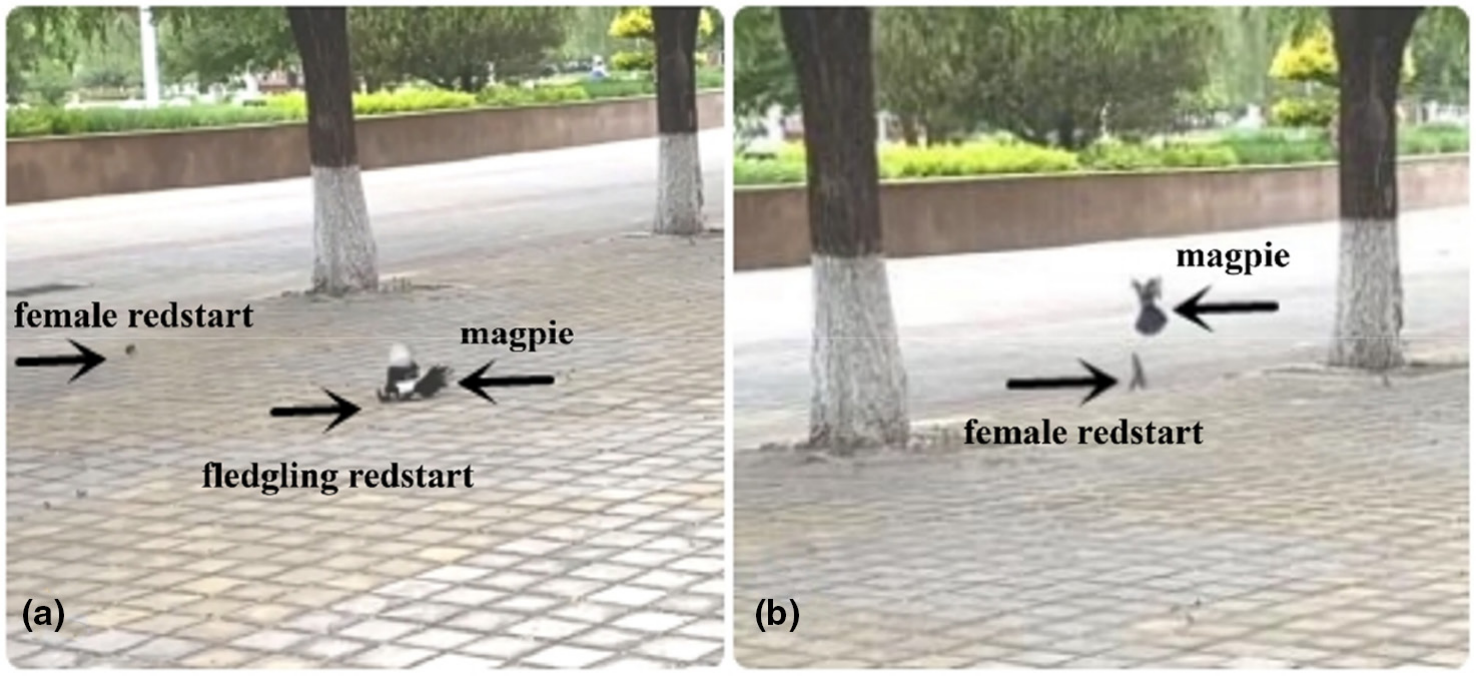
Predation on fledgling by magpie and defence by female of Daurian redstart. (a) Refers to predation on fledgling by magpie. (b) Refers to defence by female Daurian redstart.

There are about three pairs of Daurian redstart breeding with rubbish bin or artificial nest box, while Magpies are relatively common nesting above the tall trees in the campus of the Hebei Normal University for Nationalities in Chengde, Hebei Province (40°53′9″–40°53′44″ N, 117°56′48″–117°57′13″ E). A case of a magpie successfully preying on the fledgling of a Daurian redstart was recorded at 6:40 on 26 May 2021 in the campus, which was an incidental record in routine observations. (Figure 1a). The Daurian redstart fledgling was approximately 13 days old after hatching and was bred in a rubbish bin next to the teaching building on the campus. This was the first day for chicks left the nest and moved on the ground about 10 m away from the nest following their parent, which they still needed to be cared for by the parents at this stage. A magpie swooped down on and tried to hunt a chick, but it failed on its first predation because of defence by female adult bird. Then, the magpie made a quick second attempt and successfully depredated a chick and pinned it to the ground with claws. It then delivered several blows and flew off carrying the fledgling with beak. The female parent gave a fierce alarm when the fledgling was preyed on and continuously attacked and pursued the magpie to a distance of 20 m (Figure 1b). However, the latter still predated successfully, and no male parent bird was seen during the predation process. We were conscious of the first failed hunt. Then, we immediately shot with the mobile phone (iPhone 11 by Zheng and Lyu listed in the author) and recorded the second predation successfully (see Video S1 file for more details).

## 3 DISCUSSION

Compared with a fixed breeding nest, fledglings can freely move around with their parents during the post‐fledging period, rendering this stage significantly more difficult to observe and study. Predation is not only an important factor for eggs or chicks from the nest (Fu et al., 2016; Guppy et al., 2017; Yin et al., 2023) but also already known as the primary source of fledging mortality despite the identification of predators is mainly based on indirect traces after predation (Haché et al., 2014; King et al., 2006; Sullivan, 1989; but see Earsom, 2004). This study found that a post‐fledgling of the Daurian redstart was successfully predated by a magpie, which provides direct evidence that predation is an important threat to the survival of fledglings during the post‐fledging period.

Magpies are known as opportunistic foragers with catholic diets including eggs, chicks and even small adult birds, although this is extremely rare (Hendricks & Hendricks, 2022). This predation event may be due to the relative overlap of breeding area. Magpies are common in campus with abundant artificiality and buildings leading to low concealability, and the post‐fledglings have limited and weaker ability to escape risks.

The high cost of predation has led parent birds to evolve diverse antipredation strategies, such as choosing low‐risk and secluded nesting sites before breeding (MacDonald et al., 2016; Zanette et al., 2011), making alarming sounds to warn and protect their offspring when at risk of predation (Caro, 2005), imitating more aggressive types of sounds to scare off predators (Liu & Liang, 2022; Zub et al., 2017) and even direct attacks (Kryštofková et al., 2011; Ma, Yang, & Liang, 2018; Ma, Yang, Liu, et al., 2018). However, as some predators also prey on small birds, many small birds will avoid direct attacks on predators to avoid the risk of their own predation (Kleindorfer et al., 2005). The Daurian redstart observed in this study was much smaller than the magpie predator, but the parent displayed strong aggressive and repelling behaviour. This may be because the post‐fledging period was at the end of the breeding season and the parent had invested a lot of energy in raising its chick. Furthermore, offsprings are more valuable with age and getting closer to independence, so stronger defensive behaviour shown by adult bird would be consistent with parental investment theory (Shew et al., 2016) and offspring value hypothesis (Smith, 1977), or it could also be that the Daurian redstart itself had extremely strong defensive behaviour. However, in the end, the parent bird was not successful in its defence, possibly due to the large difference in size. Further research is needed to study the defensive behaviour of Daurian redstarts.

## AUTHOR CONTRIBUTIONS

**Guofen Zhu:** Methodology (lead); writing – original draft (lead). **Meinuo Zheng:** Formal analysis (equal); investigation (equal). **Shanshan Lyu:** Formal analysis (equal); investigation (equal). **Laikun Ma:** Conceptualization (lead); formal analysis (equal); writing – review and editing (lead).

## ACKNOWLEDGEMENTS

We thank Hebei Normal University for Nationalities for the support and permission to carry out this study. We would like to thank the referees for their constructive comments on this manuscript

## FUNDING INFORMATION

This work was funded by the S&T Program of Chengde (202102A068 to G.Z. and 202002A088 to L.M.).

## CONFLICT OF INTEREST STATEMENT

The authors declare that they have no competing interests.

## Supporting information


Video S1:

## Data Availability

The Video and data related to this study are available from the corresponding author upon reasonable request. Data available on request from the authors.
