# Supplementary material for: Report of a magpie preying on a post‐fledgling Daurian redstart
Source: Ecol Evol. 2023 Aug 9;13(8):e10412. doi: 10.1002/ece3.10412 (PMC10410626; doi:10.1002/ece3.10412)
Supplement: Supplementary file 1 — Video S1: [file ECE3-13-e10412-s001.zip › ece310412-sup-0001-supinfo.docx]

The video which a Common magpie (Pica pica) preying on a fledgling of Daurian redstart (Phoenicurus auroreus) was incidentally recorded by mobile phone at 6:40 on 26 May 2021. A Common magpie swooped down on and successfully depredated a chick and pinned it to the ground with claws. It then delivered several blows and flew off carrying the fledgling with beak. The female parent gave a fierce alarm when the fledgling was preyed on and continuously attacked and pursued the Common magpie to a distance of 20m. However, the latter still predated successfully, even though it failed the first time because of defence by female adult bird. We were conscious of the first failed hunt. Then we immediately shot with the mobile phone (Iphone 11) and recorded the second predation successfully.
